# Will the Bacteria Survive in the CeO_2_ Nanozyme-H_2_O_2_ System?

**DOI:** 10.3390/molecules26123747

**Published:** 2021-06-19

**Authors:** Weisheng Zhu, Luyao Wang, Qisi Li, Lizhi Jiao, Xiaokan Yu, Xiangfan Gao, Hao Qiu, Zhijun Zhang, Wei Bing

**Affiliations:** 1 Key Laboratory of Surface & Interface of Polymer Materials of Zhejiang Province, Department of Chemistry, Zhejiang Sci-Tech University, Hangzhou 310018, China; 201930107026@mails.zstu.edu.cn (W.Z.); 202020104127@mails.zstu.edu.cn (Q.L.); 202030107241@mails.zstu.edu.cn (L.J.); xiaokanyu36@gmail.com (X.Y.); g3522166853@gmail.com (X.G.); qiuhao@ciac.ac.cn (H.Q.); 2 School of Chemistry and Life Science, Changchun University of Technology, 2055 Yanan Street, Changchun 130012, China; wluyao1206@gmail.com; 3 Advanced Institute of Materials Science, Changchun University of Technology, 2055 Yanan Street, Changchun 130012, China

**Keywords:** nanozymes, cerium oxide nanoparticles, antibacterial, peroxidase, ROS scavenger

## Abstract

As one of the nanostructures with enzyme-like activity, nanozymes have recently attracted extensive attention for their biomedical applications, especially for bacterial disinfection treatment. Nanozymes with high peroxidase activity are considered to be excellent candidates for building bacterial disinfection systems (nanozyme-H_2_O_2_), in which the nanozyme will promote the generation of ROS to kill bacteria based on the decomposition of H_2_O_2_. According to this criterion, a cerium oxide nanoparticle (Nanoceria, CeO_2_, a classical nanozyme with high peroxidase activity)-based nanozyme-H_2_O_2_ system would be very efficient for bacterial disinfection. However, CeO_2_ is a nanozyme with multiple enzyme-like activities. In addition to high peroxidase activity, CeO_2_ nanozymes also possess high superoxide dismutase activity and antioxidant activity, which can act as a ROS scavenger. Considering the fact that CeO_2_ nanozymes have both the activity to promote ROS production and the opposite activity for ROS scavenging, it is worth exploring which activity will play the dominating role in the CeO_2_-H_2_O_2_ system, as well as whether it will protect bacteria or produce an antibacterial effect. In this work, we focused on this discussion to unveil the role of CeO_2_ in the CeO_2_-H_2_O_2_ system, so that it can provide valuable knowledge for the design of a nanozyme-H_2_O_2_-based antibacterial system.

## 1. Introduction

Nanozymes, as a type of nanomaterial with enzyme-like activities, have recently garnered considerable researchers’ attention toward their biological and biomedical applications [1,2,3]. Thus far, various types of nanomaterials have been reported to have catalytic activities similar to those of peroxidase, catalase, superoxide dismutase, oxidase, and other natural enzymes, including different metal and metal oxide nanoparticles, carbon-based nanomaterials, as well as some organic nanostructures [4,5,6,7]. Compared with natural enzymes, nanozymes exhibit several advantages such as tunable size and activity, facile preparation, low cost, and high stability against denaturing [8,9]. These superior properties sufficiently meet the demands for diverse biological and biomedical applications. Indeed, nanozymes have recently been frequently used for disease treatment, such as cancer therapy, bacterial disinfection, as well as Alzheimer’s disease treatment [10,11,12].

Bacterial infection has long been a major threat for human health. Antibiotics treatment is currently a golden standard for bacterial disinfection. However, the overuse of antibiotics greatly promotes antibiotic resistance development in bacterial pathogens. Therefore, the exploration of new antimicrobial strategies is urgently demanded [13,14,15,16]. Nanozyme-based antibacterial strategies have been recently demonstrated to be a promising new approach for bacterial infection treatment [17,18,19,20,21,22,23,24]. For example, Qu et al. developed graphene quantum dots (GQDs) that can act as a peroxidase mimic to enhance the antibacterial capacity of H_2_O_2_ and can be used for wound disinfection [25]. Wang and coworkers reported Cu_2_WS_4_ nanocrystals (CWS NCs) with excellent antibacterial activity owing to their high intrinsic peroxidase activity that can catalyze the decomposition of H_2_O_2_ to form ·OH, so that it inhabits wound bacterial infection [26]. Besides, Qu and his team also proposed that MOF/Ce-based nanozymes with dual enzymatic activities can not only disperse biofilms ascribed to the Ce complex, but also kill bacteria on-site, thereby avoiding the proliferation of bacteria and the recurrence of biofilms [27]. Notably, most of these works were based on the peroxidase activity of nanozymes to promote the reactive oxygen species (ROS) generation of H_2_O_2_ to kill bacteria [28,29,30,31,32,33,34]. Thus, the nanozyme with high peroxidase activity can be regarded as an excellent candidate for building a bacterial disinfection nanozyme system.

**Scheme 1:**
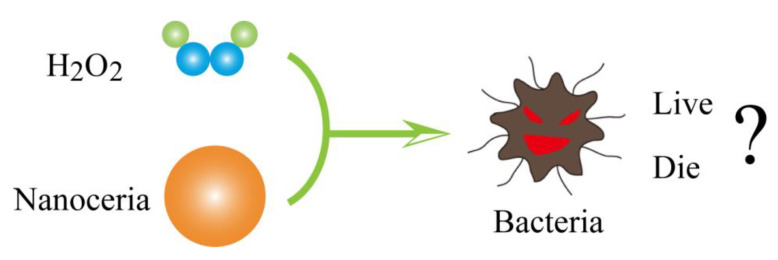
A schematic representation of the question to be answered in this work.

The cerium oxide nanoparticle (Nanoceria, CeO_2_) is a classical nanozyme with high peroxidase activity [35,36]. According to the criterion that high peroxidase activity is more conducive to promoting the production of ROS, the CeO_2_-H_2_O_2_ nanozyme system would be very efficient for bacterial disinfection. However, CeO_2_ could show multiple enzyme-like activities owing to different shapes and sizes [8,37]. In addition to high peroxidase activity, CeO_2_ also possesses high superoxide dismutase activity and antioxidant activity, which can be used as a ROS scavenger. In view of the fact that CeO_2_ has both the activity of promoting ROS production and the opposite activity of ROS scavenging, it is meaningful to discuss which activity will play the dominating role in the CeO_2_-H_2_O_2_ system, and whether it will protect bacteria or produce an antibacterial effect (Scheme 1). Herein, we focus on this discussion to unveil the role of CeO_2_ in the CeO_2_-H_2_O_2_ system, which will offer valuable knowledge for the design of a nanozyme-H_2_O_2_ based antibacterial system.

## 2. Results

### 2.1. Preparation and Characterization of CeO_2_ Nanozyme

**Figure 1:**
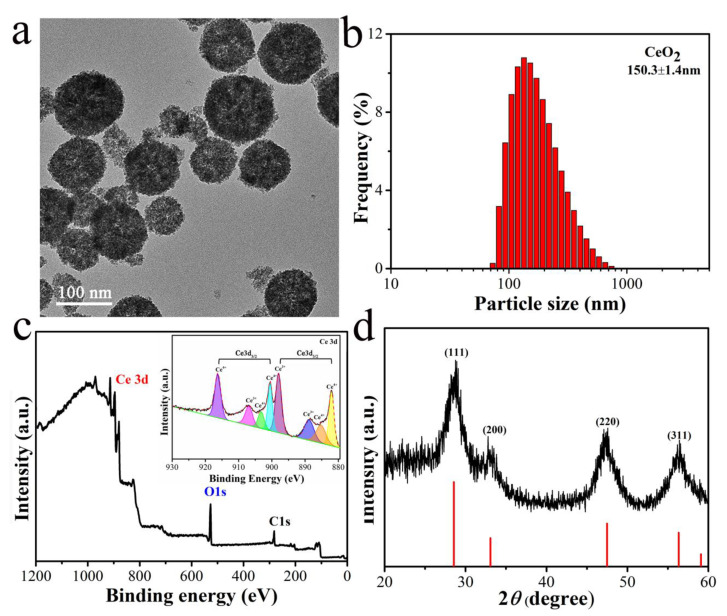
The characterization of CeO_2_ nanozyme. (**a**) TEM images of CeO_2_; (**b**) the dynamic light scattering (DLS) analysis of CeO_2_; (**c**) the XPS spectra of the CeO_2_ nanozyme; the inset shows the high-resolution XPS spectra of Ce3d; (**d**) the XRD pattern of CeO_2_ (black curve).

The CeO_2_ nanozyme was first synthesized according to a facile solvothermal method, of which the inorganic salt Ce (NO_3_)_3_·6H_2_O and organic acid C_2_H_5_COOH were selected as the initial materials without any surfactants [27]. As shown in Figure 1a, the transmission electron microscopy (TEM) images demonstrated the formation of CeO_2_ nanospheres with uniform dispersion. Dynamic light scattering (DLS) analysis was performed to determine the distribution of the hydration particle size of CeO_2_, which is placed at around 150 nm from Figure 1b. Furthermore, the X-ray photoelectron energy spectrum was employed to analyze the element and chemical value state of CeO_2_ nanospheres. We can observe from Figure 1c that there were main peaks such as Ce^4+^ 3d_3/2_ and Ce^4+^3d_5/2_ at around 914.2 and 896.0 eV in the Ce 3d core spectrum, and the Ce^3+^ 3d_3/2_ and Ce^3+^ 3d_5/2_ peaks are subscribed at 898.4 and 880.0 eV, respectively. In addition, the crystalline and phase information were implied by powder X-ray diffraction (XRD) patterns. As shown in Figure 1d, there were four clearly specific peaks, and the peaks at 2θ = 28.5°, 32.8°, 47.4°, and 56.4° can be subscribed to the characteristic (111), (200), (220), and (311) reflections of the face-centered cubic structure of CeO_2_ nanocrystals (JCPDS No. 43–1002, the red line in Figure 1d). All the above results demonstrate the successful synthesis of the CeO_2_ nanoparticles.

### 2.2. The Peroxidase Activity of CeO_2_ Nanozyme

**Figure 2:**
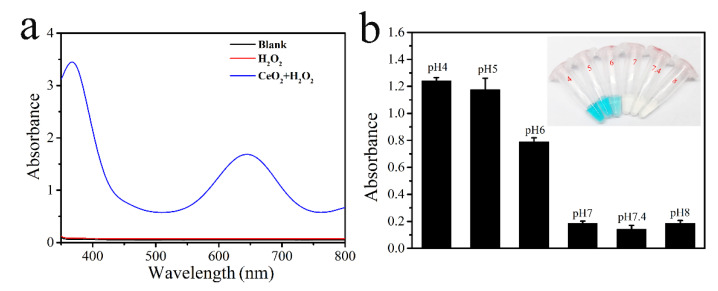
The assay of peroxidase activity of CeO2. (**a**) The absorbance of TMB at 652 nm right in different reaction systems; (**b**) the effect of different pH on absorbance of TMB at 652 nm.

In this work, we utilized a classical colorimetric assay to estimate the peroxidase activity of CeO_2_ nanospheres. TMB (3,3’,5,5’-tetramethylbenzidine) was used as a substrate. In the presence of peroxidase mimics and H_2_O_2_, colorless TMB can be oxidized to blue oxTMB, which exhibits two specific absorbance peaks at 370 and 652 nm. As shown in Figure 2a, neither the nanozyme nor H_2_O_2_ alone could produce any color changes, but there appeared two sharp and strong peaks at 370 and 652 nm with the presence of both CeO_2_ and H_2_O_2_, which indicated that CeO_2_ nanospheres had intrinsic peroxidase enzymatic activity. Likewise, we investigated the effect of pH on its peroxidase activity, and the nanozyme was demonstrated to possess optimistic catalytic activity at a pH range from 4.0 to 6.0 (Figure 2b).

### 2.3. ROS Generation

**Figure 3:**
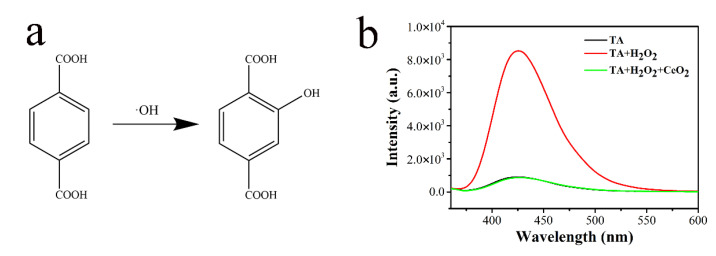
(**a**) Reaction between hydroxyl radical (OH) and terephthalic acid (TA); (**b**) fluorescence spectra of reaction system including only TA; TA and H_2_O_2_; TA, CeO_2_, and H_2_O_2_ after 2 h of reaction. [TA] = 0.5 mM, H_2_O_2_ = 1 mM, [CeO_2_] = 0.25 mg/mL.

Considering that the catalytic capacity of most of the nanozymes with peroxidase activity may originate from the decomposition of H_2_O_2_ to generate ROS hydroxyl radicals (·OH), we further investigated the generation of ·OH in the CeO_2_-H_2_O_2_ system to verify the peroxidase activity of the CeO_2_ nanozyme. Terephthalic acid (TA), which could capture ·OH to generate fluorescent product 2-hydroxy terephthalic acid (TAOH) (Figure 3a), was used as a fluorescence probe for the tracing of ·OH. As shown in Figure 3b, compared with the fluorescent intensity of TA alone, there was a remarkable increase with the addition of H_2_O_2_. However, when we added the CeO_2_ nanozyme to the H_2_O_2_-TA system, the fluorescent intensity did not show any increase but sharply decreased to near the background intensity, which was contrary to what we predicted. This may be because CeO_2_ possesses certain antioxidant properties as obstacles to the decomposition of H_2_O_2_ to generate ·OH [37]. In other words, CeO_2_ shows considerable ROS scavenging capacity in the CeO_2_ nanozyme-H_2_O_2_ system.

### 2.4. The Evaluation of Antibacterial Activity of CeO_2_-H_2_O_2_ System

**Figure 4:**
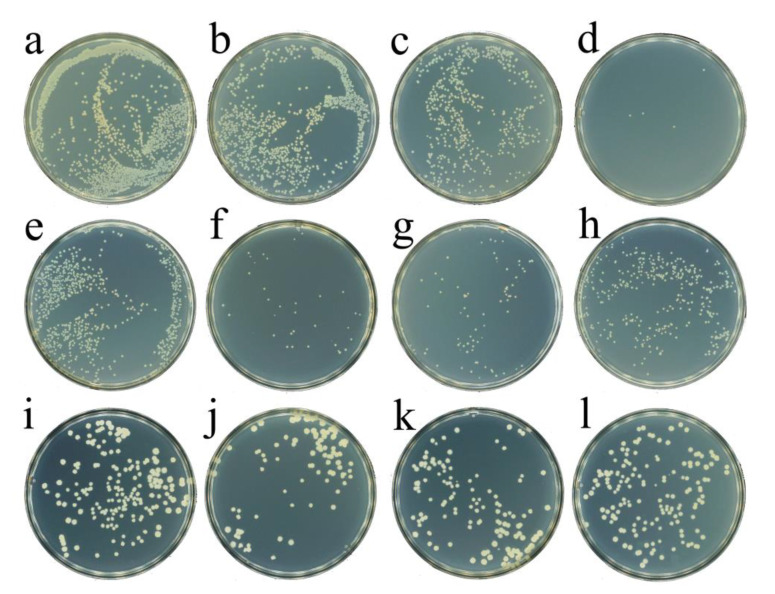
Photographs of bacterial colonies formed by *E. coli* and *B. subtilis* with different treatment at room temperature for 2 h. (**a**) *E. coli*; (**b**) *E. coli* + 0.25 mg/mL CeO_2_; (**c**) *E. coli* + 0.5 mM H_2_O_2_; (**d**) *E. coli* + 1.5 mM H_2_O_2_; (**e**) *E. coli* + 0.5 mM H_2_O_2_ + 0.25 mg/mL CeO_2_; (**f**) *E. coli* + 1.5 mM H_2_O_2_ + 0.1 mg/mL CeO_2_; (**g**) *E. coli* + 1.5 mM H_2_O_2_ + 0.25 mg/mL CeO_2_; (**h**) *E. coli* + 1.5 mM H_2_O_2_ + 0.4 mg/mL CeO_2_; (**i**) *B. subtilis*; (**j**) *B. subtilis* + 1 mM H_2_O_2_; (**k**) *B. subtilis* + 1 mM H_2_O_2_ + 0.25 mg/mL CeO_2_; (**l**) *B. subtilis* + 1 mM H_2_O_2_ + 0.4 mg/mL CeO_2_.

Finally, the antibacterial activities were investigated by using a Gram-negative bacterium (*Escherichia coli*, *E. coli*) as a model strain. In view of the effect of the pH value on the nanozyme activity, all the bacterial experiments were performed at pH 6.0 with PBS (phosphate-buffered saline) as the buffer. As shown in Figure 4b, CeO_2_ (0.25 mg/mL) alone caused a weak antibacterial effect. H_2_O_2_ showed concentration-dependent antibacterial activity. At a low concentration of 0.5 mM, H_2_O_2_ showed a weak antibacterial activity (Figure 4c). When the concentration of H_2_O_2_ was increased to 1.5 mM, almost all the bacteria were killed, indicating a significant high antibacterial activity (Figure 4d). Thereafter, the bacterial effect of the CeO_2_ nanozyme-H_2_O_2_ system was investigated. As shown in Figure 4e, compared with H_2_O_2_ (0.5 mM) alone, the presence of CeO_2_ (0.25 mg/mL) did not cause an obvious change in the number of bacteria, which demonstrated that the CeO_2_ nanozyme did not promote the killing effect of H_2_O_2_ against bacteria in this nanozyme-H_2_O_2_ system. Interestingly, compared with H_2_O_2_ (1.5 mM) alone, the presence of CeO_2_ (0.25 mg/mL) caused an obvious increase in the number of bacteria, which indicated that the CeO_2_ greatly inhibited the killing effect of H_2_O_2_ against bacteria in this nanozyme-H_2_O_2_ system (Figure 4g). This role was further found to be in a concentration-dependent manner. When we reduced the CeO_2_ concentration to 0.1 mg/mL, the effect of CeO_2_ on the bacterial killing activity of H_2_O_2_ decreased accordingly (Figure 4f). On the contrary, the effect of CeO_2_ on the bacterial killing activity of H_2_O_2_ was dramatically increased with the increase in the concentration of CeO_2_ to 0.4 mg/mL. The further study demonstrated that CeO_2_ played a similar protection role in the nanozyme-H_2_O_2_ system toward the Gram-positive bacterium (*Bacillus subtilis*, *B. subtilis*) (Figure 4f–i). All the above results manifested that the peroxidase activity of CeO_2_ did not contribute to the antibacterial effect of the nanozyme-H_2_O_2_ system. Instead, the ROS scavenging capacity of CeO_2_ protected the bacteria from being killed by H_2_O_2_. Although the phenomenon we observed may be different from other enzymes with multiple activities, our work would provide valuable new knowledge for the design of the nanozyme-H_2_O_2_-based antibacterial system.

## 3. Materials and Methods

### 3.1. Chemicals and Instrument

All reagents and solvents were purchased from commercial sources. Hydrogen- peroxide is an AR reagent with a concentration of 30% in water. The degree of purity of other compounds was at least 98%, and they were used without any further treatment.

PL spectra were collected by a FL-970 Fluorescence Spectrometer (slit width of 2.5 nm and PMT voltage of 600 V). UV-vis adsorption spectra were measured by a UV-2600 spectrometer (SHIMADZU, Japan). The size of nanoparticles was monitored with a SZ-100V2 Nano Particle Analyzer. The X-ray diffraction (XRD) patterns of the products were determined on a DX-2700 X-ray diffractometer with Cu Kα radiation (λ = 1.5416 Å), with an operation voltage and current maintained at 35 kV and 25 mA, respectively. X-ray photoelectron spectroscopy (XPS) measurements were conducted on a Kratos AXIS Ultra DLD photoelectron spectrometer with Al Kα X-ray radiation as the X-ray source for excitation. The nanostructure of products was analyzed with a 120 kV JEM-1400Flash transmission electron microscope (TEM) with a Gatan Rio16 digital camera. Samples for TEM were prepared by dropping dilute solutions of nanoparticles onto carbon-coated copper grids and then maintained at 40 °C to wait for the solvent to evaporate.

### 3.2. Synthesis of CeO_2_ Nanozyme

The CeO_2_ nanozyme was synthesized by one simple solvothermal method right in this work. Briefly, 1 g of Ce(NO_3_)_3_·6H_2_O was dissolved in 1 mL of ultrapure water. Then, 1 mL of C_2_H_5_COOH and 30 mL of glycol were added in the above solution with stirring until the formation of a uniform solution. Following that, the mixture solution was transferred to an autoclave and heated to 180 °C for 200 min to obtain the initial product. The product was first centrifuged at 5000 rpm for 10 min to remove large particles; then, the supernatant was centrifuged at 10,000 rpm for 10 min, and the precipitation was washed with ethanol and ultrapure water twice. Ultimately, the product was redispersed into ultrapure water for later use.

### 3.3. Detection of Peroxidase Activity of CeO_2_

The peroxidase activity was investigated by a catalytic reaction of the TMB with the assistance of H_2_O_2_, and the catalytic performance was characterized by a specific peak at 652 nm. Typically, the reaction solution (200 μL) containing PBS buffer solution (0.2 M, 20 μL), TMB (80 mM, 2 μL), H_2_O_2_ (10 mM, 40 μL), and CeO_2_ nanozyme (40 μL) was incubated at room temperature for 10 min, and the blue product (oxTMB) was then monitored by a UV-spectrum at 652 nm.

### 3.4. Detection of OH

The detection of ·OH was dependent on the reaction of TA and ·OH. TA has weak fluorescence in the absence of ·OH, but it has unique fluorescence around 425 nm in the presence of ·OH ascribed to the generation of 2-hydroxy terephthalic acid.

The reaction solutions containing TA, TA + H_2_O_2_, and TA + H_2_O_2_ + nanozymes were reacted for 2 h and then centrifuged to separate the supernatant. The fluorescent intensity of the supernatant at around 430 nm was determined by a FL-970 fluorescence spectrometer. The concentrations of TA, H_2_O_2_, and CeO_2_ nanozyme were 0.5 mM, 1 mM, 0.25 mg/mL, respectively.

### 3.5. Antibacterial Experiment

The spread plate method was utilized to measure the bacterial number under different treatments. *E. coli* was coped with five different groups: (a) without any addition, (b) CeO_2_, (c) H_2_O_2_, and (d) CeO_2_ + H_2_O_2_ groups. In brief, the mono-colony of *E. coli* on a solid Luria–Bertani (LB) agar plate was diverted to 2 mL of liquid LB culture medium and grown at 37 °C for 5 h under 180 rpm rotation. After finishing this process, we chose 0.3 as the initial optical density of bacteria at OD_600 nm_. Then, 20 μL of as-prepared bacteria solution was mixed up with different groups we mentioned above with a 200 μL total volume and incubated in 24-cell culture plates at a reaction system (pH = 5, PBS buffer solution 20 mM) for 2 h. Thereafter, the bacteria solution was transferred from the 24-well plate to the solid medium by the spread plate method and was cultured at 37 °C for 12 h. A similar method was used in the *B. subtilis* experiment.

## 4. Conclusions

In summary, we successfully synthesized a CeO_2_ nanozyme with both the peroxidase and ROS scavenging activity. Based on this, we investigated the function between the CeO_2_-H_2_O_2_ system and bacteria. The results of the bacterial experiments demonstrated that the peroxidase activity of CeO_2_ did not contribute to the antibacterial effect in the CeO_2_-H_2_O_2_ system; instead, the ROS scavenging capacity of CeO_2_ could protect the bacteria from being killed by H_2_O_2_. Our present work not only unveils the role of CeO_2_ in the CeO_2_-H_2_O_2_ system toward bacteria but also provides valuable new knowledge for the design of nanozyme-H_2_O_2_-based antibacterial systems.

## Data Availability

Data are contained within the article.

## Sample Availability

Samples of the compounds are available from the authors.
